# Hexa­aquadodeca-μ_2_-chlorido-*octa­hedro*-hexa­niobium diiodide

**DOI:** 10.1107/S2414314621006969

**Published:** 2021-07-20

**Authors:** Florian Schröder, Martin Köckerling

**Affiliations:** a Universität Rostock, Institut für Chemie, Anorganische Festkörperchemie, Albert-Einstein-Str. 3a, D-18059 Rostock, Germany; Goethe-Universität Frankfurt, Germany

**Keywords:** crystal structure, cluster, niobium, iodide

## Abstract

The title compound, [Nb_6_Cl_12_(H_2_O)_6_]I_2_, consists of the niobium cluster cation [Nb_6_Cl_12_(H_2_O)_6_]^2+^ and two non-coordinating, charge-balancing iodide ions. The edges of the Nb_6_ octa­hedron are bridged by chlorido ligands. Each Nb atom is further coordinated by a water ligand. The cluster cation has a charge of +2, which is balanced by that of two iodide anions.

## Abstract 2

The crystal structure of the cluster complex [Nb_6_Cl_12_(H_2_O)_6_]I_2_ is described. The octa­hedral Nb_6_ cluster core is coordinated by twelve chlorido and six water ligands. The two iodide ions are not bonded to the cluster unit but are present in the crystal structure as the counter-anion to the cluster cation.

## Graphical abstract

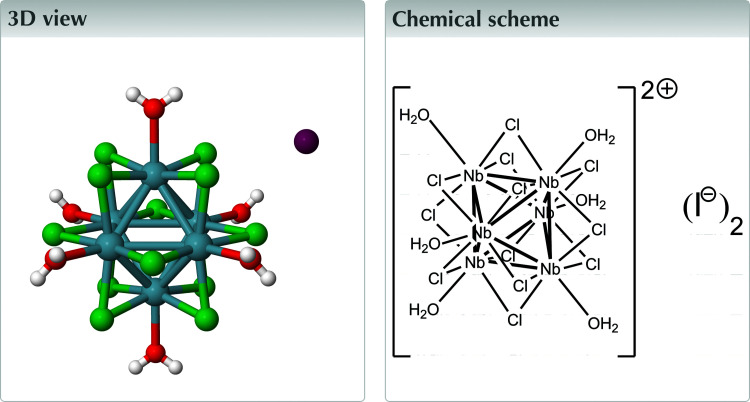

## Structure description

Cluster complexes of transition metals have been an inter­esting research area for many years (Cotton, 1964 ▸; Simon, 1988 ▸). Ligand-exchange reactions in solvents have opened up a wide field of new cluster compounds (Lemoine *et al.*, 2019 ▸), of which so far iodides have been investigated much less than chlorides.

**Figure 1:**
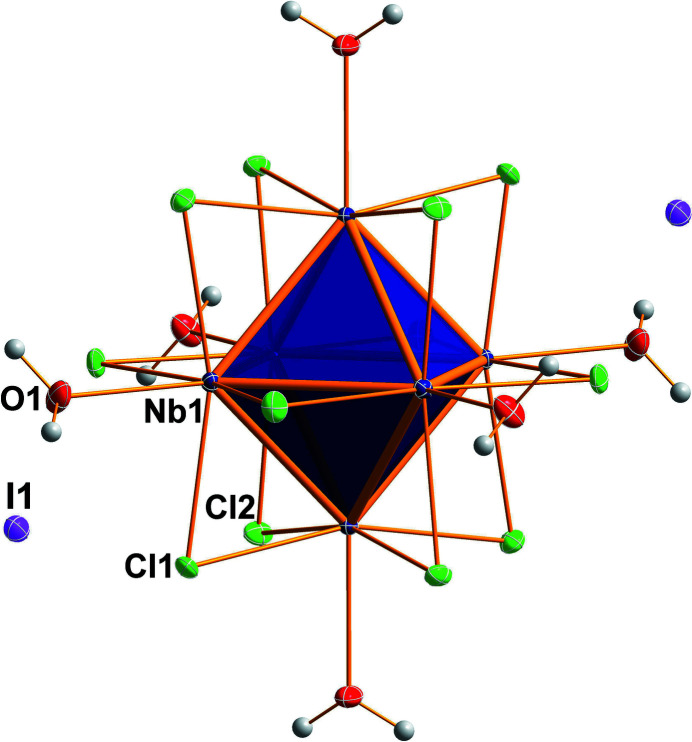
Perspective view of the title compound with atom labelling for the asymmetric unit. Displacement ellipsoids are shown at the 50% probability level.

The title compound crystallizes in the trigonal space group *P* 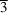 1*m*. The asymmetric unit consists of an [NbCl_2_(H_2_O)]_0.5_ unit, which is located close to the Wyckoff site 1*a* with 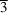 *m* symmetry, and one-sixth of an iodide ion. The Nb_6_ unit is a metal atom octa­hedron with an Nb—Nb bond length of 2.8960 (4) Å. The twelve μ_2_ bridging positions of the inner ligand sphere are occupied by chlorido ligands. An average Nb—Cl bonding length of 2.456 Å and an average Nb—Cl—Nb angle of 72.31° are present. The six positions of the outer ligand sphere are occupied by water ligands, reaching Nb—O bond lengths of 2.250 (2) Å. The structure of the cluster cation and the packing are shown in Figs. 1 ▸ and 2 ▸. The charge of the two iodide anions are counter-balanced by that of the doubly positive charged cluster cation [Nb_6_Cl_12_(H_2_O)_6_]^2+^. Based on the ion ratio and Nb—Nb bond lengths of comparable structures, 16 cluster-based electrons (CBE) are present. Even though six water molecules are present per formula unit, hydrogen bonding is essentially not present in crystals of the title compound, because the large iodide anions separate the cluster units such that the shortest O⋯O distance is 4.432 (2) Å. The only weak hydrogen-type bonding contact exists between I1 and O1 with an O1—H1*A*⋯O1 distance of 3.501 (1) Å.

## Synthesis and crystallization

Starting from the compound [Nb_6_Cl_12_I_2_(H_2_O)_4_]·4H_2_O (Schäfer *et al.*, 1972 ▸; Brnicevic *et al.*, 1981 ▸), the compound [Nb_6_Cl_12_(H_2_O)_6_]I_2_ can be synthesized in acceptable yields.

Amounts of 100 mg (72.42 µmol) of [Nb_6_Cl_12_I_2_(H_2_O)_4_]·4H_2_O and 100 mg (667.16 µmol) of NaI were dissolved in 8 ml (444.07 mmol) of degassed water at room temperature and then filtered. The obtained dark-green solution was evaporated in a crystallizing shell for 4 d. Large black single crystals were obtained in remnants of NaI. After washing several times with acetone, 65.0 mg (48.34 µmol, yield: 65%) of [Nb_6_Cl_12_(H_2_O)_6_]I_2_ were obtained. The cluster compound was further characterized as follows: Elemental analysis: *M* [H_12_Cl_12_I_2_O_6_Nb_6_] = 1344.764: found H = 1.01% (calc. 0.90%); ^1^H NMR: (MeCN-*d*_3_ was refluxed for several hours with CaH_2_ and finally distilled under Schlenk conditions) (MeCN-*d*_3_, 300 MHz, 300 K, p.p.m.): δ = 2.14 (*s*, 12H, *H*_2_O); IR (300 K, ATR, cm^−1^): ν = 406 (*s*), 600 (*s*), 692 (*s*), 1587 (*vs*), 3140 (*s*), 3256 (*s*).

## Refinement

Crystal data, data collection and structure refinement details are summarized in Table 1 ▸. Two reflections (001 and 010) were omitted from the structure refinement because their intensities were affected by the beam stop.

## Supplementary Material

Crystal structure: contains datablock(s) I. DOI: 10.1107/S2414314621006969/bt4115sup1.cif

Structure factors: contains datablock(s) I. DOI: 10.1107/S2414314621006969/bt4115Isup2.hkl

CCDC reference: 2094789

Additional supporting information:  crystallographic information; 3D view; checkCIF report

## Figures and Tables

**Figure 2:**
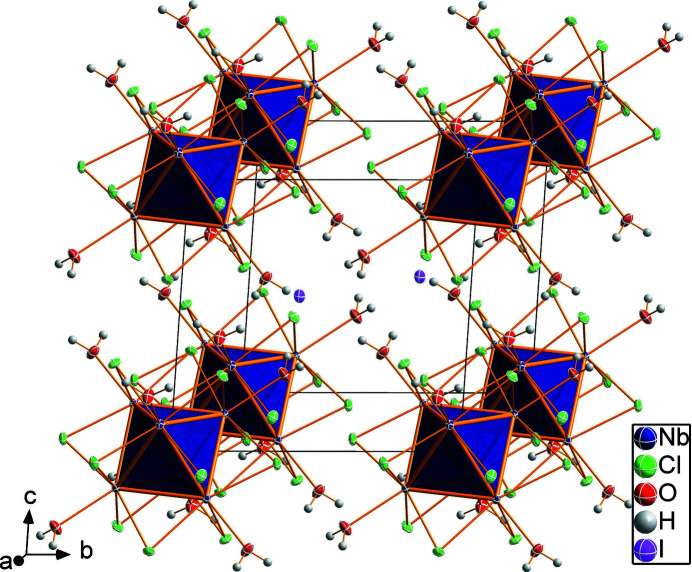
Packing of the cluster cations and iodide anions in the crystal of the title compound.

**Table 1:** Experimental details

Crystal data	
Chemical formula	[Nb_6_Cl_12_(H_2_O)_6_]I_2_
*M*_r_	1344.76
Crystal system, space group	Trigonal, *P* 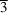 1*m*
Temperature (K)	123
*a*, *c* (Å)	9.3911 (8), 8.6576 (9)
*V* (Å^3^)	661.2 (1)
*Z*	1
Radiation type	Mo *K*α
μ (mm^−1^)	6.08
Crystal size (mm)	0.20 × 0.20 × 0.16

Data collection	
Diffractometer	Bruker APEXII CCD
Absorption correction	Multi-scan (*SADABS*; Bruker, 2017 ▸)
No. of measured, independent and observed [*I* > 2σ(*I*)] reflections	43489, 1059, 1058
*R*_int_	0.031
(sin θ/λ)_max_ (Å^−1^)	0.806

Refinement	
*R*[*F* ^2^ > 2σ(*F* ^2^)], *wR*(*F* ^2^), *S*	0.013, 0.034, 1.38
No. of reflections	1059
No. of parameters	27
H-atom treatment	H-atom parameters constrained
Δρ_max_, Δρ_min_ (e Å^−3^)	0.72, −0.81

Computer programs: *APEX3* and *SAINT* (Bruker, 2017 ▸), *SHELXS2014* (Sheldrick, 2008 ▸), *SHELXL2014* (Sheldrick, 2015 ▸), *DIAMOND* (Brandenburg & Putz, 2019 ▸) and *publCIF* (Westrip, 2010 ▸).

## full crystallographic data

### Crystal data

**Table:**

[Nb_6_Cl_12_(H_2_O)_6_]I_2_	*D*_x_ = 3.377 Mg m^−^^3^
*M**_r_* = 1344.76	Mo *K*α radiation, λ = 0.71073 Å
Trigonal, *P*31*m*	Cell parameters from 9268 reflections
*a* = 9.3911 (8) Å	θ = 2.5–34.9°
*c* = 8.6576 (9) Å	µ = 6.08 mm^−^^1^
*V* = 661.2 (1) Å^3^	*T* = 123 K
*Z* = 1	Block, black
*F*(000) = 616	0.20 × 0.20 × 0.16 mm

### Data collection

**Table:**

Bruker APEXII CCD diffractometer	1058 reflections with *I* > 2σ(*I*)
Radiation source: microfocus sealed tube	*R*_int_ = 0.031
φ and ω scans	θ_max_ = 35.0°, θ_min_ = 3.4°
Absorption correction: multi-scan (SADABS, Bruker, 2017)	*h* = −15→15
	*k* = −15→15
43489 measured reflections	*l* = −13→13
1059 independent reflections	

### Refinement

**Table:**

Refinement on *F*^2^	Secondary atom site location: difference Fourier map
Least-squares matrix: full	Hydrogen site location: difference Fourier map
*R*[*F*^2^ > 2σ(*F*^2^)] = 0.013	H-atom parameters constrained
*wR*(*F*^2^) = 0.034	*w* = 1/[σ^2^(*F*_o_^2^) + (0.005*P*)^2^ + 1.1433*P*] where *P* = (*F*_o_^2^ + 2*F*_c_^2^)/3
*S* = 1.38	(Δ/σ)_max_ < 0.001
1059 reflections	Δρ_max_ = 0.72 e Å^−^^3^
27 parameters	Δρ_min_ = −0.81 e Å^−^^3^
0 restraints	Extinction correction: SHELXL, Fc^*^=kFc[1+0.001xFc^2^λ^3^/sin(2θ)]^-1/4^
Primary atom site location: structure-invariant direct methods	Extinction coefficient: 0.0071 (3)

### Special details

**Table:**

Geometry. All esds (except the esd in the dihedral angle between two l.s. planes) are estimated using the full covariance matrix. The cell esds are taken into account individually in the estimation of esds in distances, angles and torsion angles; correlations between esds in cell parameters are only used when they are defined by crystal symmetry. An approximate (isotropic) treatment of cell esds is used for estimating esds involving l.s. planes.
Refinement. The H atom was located in a difference maps, but refined using a riding model with O—H = 0.85 Å and with *U*(H) = 1.5 *U*_eq_(O).

### Fractional atomic coordinates and isotropic or equivalent isotropic displacement parameters (Å^2^)

**Table:**

	*x*	*y*	*z*	*U*_iso_*/*U*_eq_	
Nb1	0.0000	0.17816 (2)	0.13652 (2)	0.00709 (5)	
Cl1	−0.21059 (3)	0.21059 (3)	0.0000	0.01203 (9)	
Cl2	0.21271 (6)	0.21271 (6)	0.32300 (6)	0.01238 (9)	
O1	0.0000	0.3770 (2)	0.2816 (2)	0.0167 (3)	
H1A	0.0851	0.4473	0.3300	0.025*	
I1	0.333333	0.666667	0.5000	0.01368 (6)	

### Atomic displacement parameters (Å^2^)

**Table:**

	*U*^11^	*U*^22^	*U*^33^	*U*^12^	*U*^13^	*U*^23^
Nb1	0.00681 (8)	0.00640 (6)	0.00821 (8)	0.00340 (4)	0.00	−0.00114 (4)
Cl1	0.0111 (1)	0.0111 (1)	0.0173 (2)	0.0082 (2)	−0.0021 (1)	−0.0021 (1)
Cl2	0.0136 (2)	0.0136 (2)	0.0106 (2)	0.0072 (2)	−0.0044 (1)	−0.0044 (1)
O1	0.0135 (7)	0.0154 (5)	0.0205 (7)	0.0067 (3)	0.00	−0.0085 (5)
I1	0.01186 (7)	0.01186 (7)	0.0173 (1)	0.00593 (4)	0.00	0.00

### Geometric parameters (Å, º)

**Table:**

Nb1—O1	2.250 (2)	Nb1—Nb1^i^	2.8960 (4)
Nb1—Cl1	2.4502 (4)	Nb1—Nb1^ii^	2.8980 (4)
Nb1—Cl1^i^	2.4502 (4)	Nb1—Nb1^iv^	2.8980 (4)
Nb1—Cl2^ii^	2.4605 (4)	Cl1—Nb1^iii^	2.4501 (4)
Nb1—Cl2	2.4605 (4)	Cl2—Nb1^iv^	2.4605 (4)
Nb1—Nb1^iii^	2.8960 (4)	O1—H1A	0.8500
			
O1—Nb1—Cl1	80.29 (4)	Nb1^iii^—Nb1—Nb1^i^	60.04 (1)
O1—Nb1—Cl1^i^	80.28 (4)	O1—Nb1—Nb1^ii^	135.94 (3)
Cl1—Nb1—Cl1^i^	88.699 (7)	Cl1—Nb1—Nb1^ii^	96.180 (6)
O1—Nb1—Cl2^ii^	82.02 (4)	Cl1^i^—Nb1—Nb1^ii^	143.773 (8)
Cl1—Nb1—Cl2^ii^	88.26 (1)	Cl2^ii^—Nb1—Nb1^ii^	53.922 (8)
Cl1^i^—Nb1—Cl2^ii^	162.30 (1)	Cl2—Nb1—Nb1^ii^	96.56 (1)
O1—Nb1—Cl2	82.02 (4)	Nb1^iii^—Nb1—Nb1^ii^	59.977 (5)
Cl1—Nb1—Cl2	162.30 (1)	Nb1^i^—Nb1—Nb1^ii^	90.0
Cl1^i^—Nb1—Cl2	88.26 (1)	O1—Nb1—Nb1^iv^	135.94 (3)
Cl2^ii^—Nb1—Cl2	89.35 (3)	Cl1—Nb1—Nb1^iv^	143.773 (8)
O1—Nb1—Nb1^iii^	134.05 (4)	Cl1^i^—Nb1—Nb1^iv^	96.180 (6)
Cl1—Nb1—Nb1^iii^	53.773 (8)	Cl2^ii^—Nb1—Nb1^iv^	96.56 (1)
Cl1^i^—Nb1—Nb1^iii^	96.23 (1)	Cl2—Nb1—Nb1^iv^	53.922 (8)
Cl2^ii^—Nb1—Nb1^iii^	96.00 (1)	Nb1^iii^—Nb1—Nb1^iv^	90.0
Cl2—Nb1—Nb1^iii^	143.919 (8)	Nb1^i^—Nb1—Nb1^iv^	59.977 (5)
O1—Nb1—Nb1^i^	134.05 (4)	Nb1^ii^—Nb1—Nb1^iv^	60.0
Cl1—Nb1—Nb1^i^	96.23 (1)	Nb1^iii^—Cl1—Nb1	72.45 (2)
Cl1^i^—Nb1—Nb1^i^	53.773 (8)	Nb1—Cl2—Nb1^iv^	72.16 (2)
Cl2^ii^—Nb1—Nb1^i^	143.919 (8)	Nb1—O1—H1A	122.0
Cl2—Nb1—Nb1^i^	96.01 (1)		

Symmetry codes: (i) *y*, −*x*+*y*, −*z*; (ii) −*y*, *x*−*y*, *z*; (iii) *x*−*y*, *x*, −*z*; (iv) −*x*+*y*, −*x*, *z*.
